# Promoting Inclusive Outdoor Recreation in National Park Governance: A Comparative Perspective from Canada and Spain

**DOI:** 10.3390/ijerph19052566

**Published:** 2022-02-23

**Authors:** Maria José Aguilar-Carrasco, Eric Gielen, Maria Vallés-Planells, Francisco Galiana, Mercedes Almenar-Muñoz, Cecil Konijnendijk

**Affiliations:** 1 Department of Urbanism, School of Civil Engineering, Universitat Politècnica de València, 46022 Valencia, Spain; egielen@urb.upv.es; 2 Department of Agrifood and Rural Engineering, School of Agricultural Engineering and Environment, Universitat Politècnica de València, 46022 Valencia, Spain; convalpl@agf.upv.es (M.V.-P.); fgaliana@agf.upv.es (F.G.); 3 Department of Urbanism, School of Architecture, Universitat Politècnica de València, 46022 Valencia, Spain; meralmuo@urb.upv.es; 4 Department of Forest Resources Management, Faculty of Forestry, University of British Columbia, Vancouver, BC V6T 1Z4, Canada; cecil.konijnendijk@ubc.ca

**Keywords:** accessibility, environmental equity, legislation, people with disabilities, public use, good governance, intersectionality, stewardship

## Abstract

While national parks (NPs) have for a long time made substantial contributions to visitor well-being, many spaces remain out of reach of people with disabilities (PwDs). This is partly due to a lack of policies that take accessibility for broader intersectional audiences into consideration. This paper evaluates governance and legal frameworks in NPs in both Canada and Spain. A decision-making framework based on intersectionality realities is proposed to assess current conditions of environmental good governance using a set of descriptors created to scrutinize laws and technical documents that can promote equitable access to NPs. To validate results derived from the regulatory evaluation, semistructured interviews with park managers were carried out. Results revealed the importance of incorporating equity discourses into policies that regulate NP networks to guarantee that all the intersectional realities for park uses are considered in their management. Furthermore, when a country develops a well-structured federal framework under which the rights of PwDs are ensured, it transcends other fields of law. Differences between the Canadian and the Spanish situation are highlighted, as well as the need for links between higher-level policies and laws and on-the-ground implementation, with NP management plans playing an important role.

## 1. Introduction

Direct contact with nature provides benefits to human health [1,2]. Natural protected areas (NPAs) are spaces of special interest due to the quality of their ecosystems, which has made them desirable places to visit [3]. According to data from the International Union for Conservation of Nature (IUCN) published in the Protected Planet Report 2012 [4], 12.7% of the world’s land and 1.6% of the world’s ocean are recognized as NPAs. Among these, national parks (NPs) are eagerly anticipated by society and have the objective of protecting large-scale ecological processes to maintain their ecosystem services and functions, which sustain human life as we know it. In addition, educational purposes are a main objective and are focused on enjoying the outdoor opportunities they provide [5,6,7,8,9,10].

Despite the fact that NPs have become a tourist attraction due to the increase in the number of visitors reported annually [3], there are still some places that exclude a significant percentage of society, such as people with disabilities (PwDs), more specifically those who have mobility/motor disabilities (PwMDs), who historically have been cut off from leisure activity in natural areas. The lack of data on PwDs in NP visits in statistics published by them demonstrates, at the very least, that they are not being considered. The fact that PwMDs enjoy less of NPs than those who are not disabled [11,12] may be for various reasons: the individual’s own decision, a lack of infrastructure and equipment facilitating their access, or not having enough information about these places [13,14,15,16].

Disabilities is an umbrella term, covering problems in body function or structure, or activity limitations, such as difficulties encountered by an individual in executing a task or action, or a restriction on participation understood as a problem experienced in involvement in life situations [17]. The main purpose of the United Nations’ 1993 Convention on the Rights of Persons with Disabilities (CRPD) was to change attitudes towards and approach to PwDs so that they are treated as “subjects” with rights capable of claiming them and making their own decisions, as well as being active members of society [17,18].The Standard Rules on the Equalization of Opportunities for Persons with Disabilities was adopted in the 85th plenary meeting on 20 December 1993, which concluded in the resolution A/RES/48/96, 4 March 1994 [19], also in the constitution at least of Western nations. Subsequently, policies of each country must regulate all concerns to guarantee PwD rights (work, education, health, leisure, etc.) so that they may subsequently be taken into consideration and applied to any other field, which may directly or indirectly affect their needs, assessing the scope of their integration thanks to active participation in decision making, so that their inclusion will be effective and efficient [14,18,20,21]. These considerations must always be subject to the primary objective of NPs, which is the protection of ecological integrity [5,6]. In this way, it would seem advisable to build frameworks from a transformative intersectional approach. This paradigm allows for a more critical focus, developing a multidirectional crossroads of interconnections of power, identity, and discrimination. Gender and other human social subjectivities, such as abilities or disabilities, are also considered [22,23,24]. In the case of PwDs, not only the lack of the environments’ accessibility, but also their participation in decision making on issues that directly affect them has not been taken into consideration [25,26,27,28].

Several authors have analyzed issues that may affect PwDs’ enjoyment of natural environments. Some are related, whether they are NPs or an urban green environment [11,29,30,31]. Newman and Park (n/i) focused on more specific aspects of the public use of NPs, introducing the outdoor recreation access route (ORAR) based on accessibility standards for outdoor activities, which have been developed from the most advanced policies in this area, the US and the UK legislation [32].

A path’s accessibility indicator has been configured through geospatial analysis [15] where wheelability and walkability are the main goal to advance in accessible configurations of wild or urban parks for PwMDs throughout their integration into outdoor activities [32]. Highlighting NPs’ educational purposes, Sugerman [33] suggested the implementation of an inclusive education model in outdoor adventure activities in parks. The involvement of actors with disabilities in decision making on NP accessibility was also recently addressed by Groulx et al. [25], who agree about the obviousness of involving a population affected by the lack of accessibility in any research and decision making on matters that directly affect them. Finally, one criterion that defines and characterizes the sustainability of natural area tourism, according to sustainable development goals (SDGs), is active outdoor participation for PwDs [10,26,27].

To date, causes that can constrain PwMD experiences when visiting green wild areas have been studied in a sectoral way. However, how these issues can be overcome has not been systematically evaluated from the point of view of whether policies enable or disable their inclusive and equitable experience to go into the woods. The existence of regulations that address inclusion (international, national, and regional) applied carefully to urban environments [34] suggests the need to address the issue systematically. Whether these regulations and their management instruments adequately integrate from the start their needs for using and enjoying NPs needs to be studied.

Therefore, it is a matter of verifying whether it is regulatory frameworks themselves, both regarding NPAs and PwDs, that are not allowing them to relate to natural environments and identifying parameters from an intersectional point of view that are conditioning them in their enjoyment of outdoor activities. This could be undertaken using a decision-making framework that must be based on their needs to predict to what extent the regulatory framework has gaps in it that impede the development of a governance that allows PwDs to enjoy nature.

The central question of this paper is how intersectionality in legal frameworks can contribute to dealing with previously neglected inequalities and thus to promoting a more inclusive and more equal enjoyment of NPs and their natural values by PwMDs. International frameworks and key references, such as [8,35,36,37], provide the basis for this evaluation. Equity inputs related to the governance, legislation, and management of NPs are examined to promote accessible environment outputs.

## 2. Materials and Methods

A decision-making framework is proposed to assess how adapted governance [35] in NP management is to the requirements established by intersectionality in terms of inclusive public use [23]. The object of this analysis are the international and national norms and the management instruments that regulate NP conservation activities at different levels of decision making [6], including the international treaty for the conservation of nature [38] and the plan management of the public use of each NP.

To validate good governance [36,39] in NP legislation in a systematic way, the framework includes a set of descriptors based on standards that include the limiting needs of PwDs in terms of accessibility to NPs. These descriptors are applied in two different countries to show similarities and divergences between their legislations. Finally, two case studies are analyzed, with data being collected through semistructured interviews [40] with park managers, to validate the results derived from the evaluation of the regulations and to explore to what extent the legislation’s philosophy is being transferred to the parks’ planning instruments.

### 2.1. Ambit of Study

The study is focused on the Canadian and Spanish legal frameworks. Both countries have signed the Convention on the Rights of Persons with Disabilities (CRPD) of the United Nations (1993), as well as the Convention on Biological Diversity (CBD) [38], which is the basis for incorporating intersectionality into regulatory frameworks. The Standard Rules on the Equalization of Opportunities for Persons with Disabilities was adopted in the 85th plenary meeting on 20 December 1993, which concluded in the resolution A/RES/48/96, 4 March 1994. It advises that states should ensure that all systems of society and the environment related to services, activities, information, and documentation are made available to all, particularly to PwDs [19]. Canada signed the CRPD on 30 March 2007, and its ratification was on 11 March 2010 [41]. Canada also ratified the Convention on Biological Diversity (CBD) on 4 December 1992 and became a party on 29 December 1993 [42]. Spain signed CRPD on 27 September 2007, ratifying it on 24 September 2009 [43], while the CBD was ratified on 21 December 1993, followed by becoming a party on 21 March 1994 [44]. It is assumed that if treaties have been ratified, federal policies and legal frameworks will be revised accordingly if needed. The legal system in Canada is based on common law, which is a system based on jurisprudence or case law, since its main source is judicial decision. Its main characteristics are: (i) there is not always a written constitution or codified laws, (ii) and judicial decisions are binding [45].

In Canada, the rights of PwDs were recognized in the Charter of Rights and Freedoms (1981), under point 15 Equality Rights, and regulated in the Canadian Human Rights Act of 1985 (CHR, 1985). The purpose of this act was to combat discrimination (Arts. 5 to 14) and to ensure a management plan with inclusive outdoor recreation at regulated NPs (Arts. 17 to 24) [46,47]. Recently, the act to ensure a barrier-free Canada, S.C. 2019, c.10, commonly known as Accessible Canada Act (ACA), which came into force in July 2019, recommends removing existing disability barriers faced by PwDs in matters coming under Canadian federal jurisdiction. In addition, the creation of new barriers should be foreseen, providing a structure that complies with accessibility standards [48].

Initially, NPs in Canada were considered places for recreation and tourism rather than for ecological protection, which later became the main objective for parks under the Canada National Parks Act of 1979 (CNPA) [49]. In 1985, the amended CNPA emphasized that public use should be regulated through each NP’s management plan according to the NP’s zoning. This will ensure the ecological integrity of natural resources, which is the most important objective to protect some areas under the CNPA [50]. NPs were considered a special type of public land administered by the federal government under the provisions of the CNPA. Parks Canada is the federal agency (within the Department of Canadian Heritage) that manages the whole Canadian system of protected natural and cultural heritage made up of NPs, national marine conservation areas, national urban parks, national historic sites, heritage buildings and townsite communities in NPs, heritage railway stations, and so forth [51,52]. To complete the NP network, each natural area should be represented by a new park. Policy frameworks were developed as the National Parks System Plan (1997). Each new amended park management plan must be approved by the federal minister in charge of NPs and tabled in parliament. According to the Parks Act Regulation, it is a constitutional requirement that NP lands be federal government property through an official agreement. Where lands are subject to a comprehensive land claim settlement by aboriginal communities (First Nations), Parks Canada will work closely with them throughout the process of founding the new park. Although the federal government regulates NPs, they all must have a management plan, which should be developed by regions and local communities in a participatory manner. Management plans have a 15-year lifespan, with reviews every 5. Each park management plan provides a park-specific roadmap for delivering the core elements of Parks Canada’s mandate, namely, visitor experience, public understanding and awareness, and heritage resource protection. Its network is represented by 39 natural areas from the Atlantic to the Pacific Ocean and Arctic coast [49,53].

Spanish legislation is based on Roman law, unlike Canadian law. These are legal norms that are written out, which means everyone has the opportunity to know them. It is a right based on sectoral law codes that enshrine basic rights and obligations [45].

The Spanish Constitution (1978), in Articles 14 and 49, promotes the absence of discriminatory practices and establishes the mandatory role of public authorities in ensuring the integration of people with disabilities. Furthermore, Spain regulates more specific PwD rights through the Royal Degree-Law 1/2013 of November 29, a general act on the rights of people with disabilities and their social inclusion [54,55].

The first Spanish NP legal framework was in 1916 with the National Park Act and the official founding of the first two NPs: Picos de Europa and Ordesa y Monte Perdido—then called Covadonga Mountain—and Ordesa Valley and Ara River National Parks. The Spanish initiative was inspired by the example of the United States’ NPs, following a romantic vision that prevailed over scientific criteria related to landscape or biodiversity [56]. This perspective also characterized Spanish NP governance as formalized in the laws of 1954, 1975, and 1989. Law 4/1989, 27 March, on the Conservation of Natural Areas, Flora, and Fauna, introduced technical planning of natural resources and transferred some of the authority to the regions [57]. This process was ratified by several decisions of the Spanish Supreme Court (102/1995, 194/2004, 101/2005, and 99/2013). According to the latter, and as confirmed in today’s National Parks Act 30/2014 of 3 December, the state has the authority to manage the National Park Master Network Plan. The Spanish regions are in charge of managing and governing NPs through their mandatory plan without any interference from the state [58], unlike in Canadian park governance. The Spanish NP network will be complete when it includes 40 representative natural systems according to the Spanish Natural Systems Annex to the law. As shown above, Spain has two important governance documents: first, the National Park Master Network Plan [59], which is the most important tool for planning and regulating NPs for a maximum of 10 years, and second, the regions in which an NP is located must develop the Public Use Plan and Management and Use Guidelines. The latter comprise the NPs’ regular planning instrument with a set of general guidelines and rules for park use and management, also with a minimum effective term of 10 years (Arts. 19 and 20) [60].

In Table 1, a brief description of both countries’ NP network is provided. It has been constructed using data from the Canada National Parks Act (S.C. 2000, c. 32) (2000) [52], Canada’s National Park System Plan (1997) [53], Parks Canada Guiding Principles and Operational Policies [61], Law 30/2014 of 3 December 2014 of Spanish National Parks [60], and Royal Decree 389/2016 of 22 October 2016, which approves the master plan of the Spanish National Parks Network [59].

Despite their differences regarding legislation and regulations for NPs and their management, both countries have signed relevant international treaties. Thus, in principle they have incorporated the philosophies of these treaties into their federal legal framework. Additionally, both countries have a consolidated NP network with similarities and differences, which are described above. Analysis of the legal framework of both countries allows us to detect dysfunctions or gaps in the standard and make proposals for regulatory improvement.

For the local-level study, Yoho National Park in British Columbia, Canada, and Aigüestortes i Estany de Sant Maurici (AESM) National Park in Catalonia, Spain, were analyzed. Both NPs cover on-land areas and have a management plan that manages the areas intended for public use. Yoho NP’s dates from 2003, and the new one is currently undergoing its approval process, while AESM NP adopted its current plan in 2010. Both NPs have bio-geo-cultural similarities, such as being part of an alpine mountain system (the Canadian Rocky Mountains and Spanish Pyrenees) and important heritage values on their lands [62,63,64]. Their mountain landscapes also represent a challenge in terms of recreational accessibility and are very popular between nature and adventure tourists [65,66].

### 2.2. Governance Analysis of NP Management

Based on governance theory and the policy arrangement approach [28,30,35,36], our methodology was developed to decide whether it is possible to reconcile the uses of NPs, considering intersectional realities, with their ecological preservation through good governance and responsible stewardship [67,68].

The framework is based on the tetrahedron model proposed by Liefferink et al. (2006) and the key principles of governance proposed by FAO (2011). The tetrahedron model proposed by Liefferink et al., (2006) as part of the policy arrangement approach, was applied as the analytical framework to undertake an in-depth evaluation guided by the inclusive NP outdoor recreation principle. It proposes evaluating policy practices according to four dimensions: discourses, rules, actors, and resources [39]. In our case, we apply those as:

- Discourses: international treaties and their philosophies;
- Rules: laws and plan management regulations;
- Actors: people or organizations with the capacity to impact decisions about the management plan and those who administrate resources, including PwDs;
- Resources: planning tools and budgets of stewardship.

These dimensions are connected to the key principles proposed by FAO (2011) to assess and monitor forest good governance [36]—effectiveness, participation, accountability, and equity/fairness.

Data from relevant literature and reports were obtained from administration home pages in both countries, and collection was completed in June 2021. The Spanish literature was complemented with directives from the European Union law from the EUR-Lex website [69]. Relevant international treaties were also downloaded from their official websites. Further information was obtained by a thematic search of online scientific databases (Web of Science, Scopus, and Google Scholar). The keywords used for this wider-scoped search included: inclusive public use, national parks, governance, stewardship, intersectionality, facilities, outdoor recreation, people with disabilities, equity, and accessibility. A wide range of publications was considered, including peer-reviewed journal articles, books, reports, conference proceedings, and policy and guideline documents.

Next, a comparative table was built with a set of 14 descriptors structured according to [39] the dimensions (Table 2). Each descriptor is also related to the FAO key principles defined above. Descriptors were constructed inspired by Director’s Order #42, accessibility for visitors with disabilities in US National Park Service programs and services [70], the UK Countryside for All Good Practice Guide [71], the EUROPARC-España (2005) manual of public use concepts [72], the EUROPARC-España (2007) catalog of best practices regarding accessibility in protected natural areas [73], the US guide to managing the NP System Plan [74], and the catalogue of good accessibility practices in natural protected areas, and taking into account the author’s experience as a PwD enjoying parks.

Data were compiled for both countries considering federal and regional NP regulations and planning tools: documents that are binding or mandatory and others that are not. All these were scrutinized using the accessibility descriptors to see whether the FAO principles were taken into consideration in the explanatory memorandum, chapters, and articles (the body or content) of the consulted documents. Voluntary or mandatory mentions of each descriptor were checked. Each indicator was then qualitatively typified according to this scale:

- A: provisions in the law, guides, or management plan statements;
- B: no specific provisions but stipulated in the law;
- C: no specific regulation;
- NA: not applicable, because not focused on wilderness experience.

The results from the legal framework and management instruments of the NP network are organized in four tables, one for each dimension [39]. The regulations and planning tools for each country are typified according to the aforementioned dimension and the scale on which they operate.

The study was then completed with a local level analysis of both countries. The objective was to evaluate how the philosophy of the federal regulation influenced park policies’ applicability in favor of improving their accessibility for PwMDs.

The park management plan provides a sound basis for studying the equity of NPs’ recreational uses, where amenities must be developed to make it possible for PwDs to enjoy nature. NP management plans were analyzed through semistructured interviews with managers from both NPs selected [40]. This part of the study was approved by the Behavioural Research Ethics Board of the University of British Columbia (Approval Certificate Number: H20-00582) and Parks Canada Agency Research and Collection Permit (Permit Number: YNP-2020-35637). The Yoho NP questionnaires and full data table are included in Appendix A. The AESM questionnaire and full data table are in Appendix B.

## 3. Results

### 3.1. Legal Framework Assessment

Ten legal documents for Canada and seven for Spain, which correspond to the documents in force on which NPs’ network management is based, were assessed. A major difference between the two countries was detected between the two park agencies. The Canadian Parks Agency is accountable of NPs and other types of heritage that are under its jurisdiction, such as national historic sites, federal heritage buildings, and townsite communities within NPs [51,52], while in Spain heritage sites are managed by other bodies. Therefore, specific acts referring to Canadian heritage managed by the Canadian Parks Agency are also included for the Canada parks legal framework.

Table 3, Table 4, Table 5 and Table 6 show the comparative results of governance for the discourses, rules, actors, and resource dimensions, respectively. Laws and regulations and guides and technical documents are separated and hierarchically organized from the national to the local level. The laws and regulations evaluated for Canada were: the Canada National Parks Act (S.C. 2000, c.32), Canada National Marine Conservation Areas Act (S.C. 2002, c,18), Parks Canada Agency Act (S.C. 1998, c. 31), Historic Sites and Monuments Act (R.S.C. 1985, c.H-4), NP General Regulations (SOR/78-213), and Heritage Railway Stations Protection Act (R.S.C. 1985, c.52, 4th Supp.) [49,75,76,77,78,79]. In terms of guidelines and management documents, the NP System Plan (1997), Parks Canada Guiding Principles and Operational Policies (PCGPOP) (2019), Parks Canada Agency Departmental Plan 2019–2020 and 2020–2021, and Yoho NP of Canada Management Plan (2010) [51,52,53,61,80] should be mentioned. For Spain, the laws and regulation analyzed were: the Spanish National Parks Act 30/2014, Natural and Biodiversity Heritage Law (42/2007, December 13, last amended 15 September 2015), and Law 7/1988 of 30 March on the reclassification of the AESM NP. Moreover, the NP Master Plan (Royal Decree 389/2016 of 22 October), Uses and Management Master Plan of the AESM Park (Decree 39/2003 of 4 February), and AESM Park’s Public Use Plan were evaluated as guidelines and management documents [59,60,81,82,83,84].

The information provided in Table 3 shows that:

1. The nature for all objective (NAO) lacks specific regulation in the Canadian laws and regulations (C). Although NAO is still not properly integrated, the PCGPOP stipulates this descriptor even though there are no specific provisions. The 2019–2020 departmental plan (with an A score) proposes a more determined commitment to access for PwDs to parks. In the Spanish case, the NP act (with an A) regulates in its fifth article the right for all to access nature, but the decree creating AESM does not include any regulation regarding this concern. Of note are the results regarding guidelines (A qualification) and management documents, which are clearly committed to favoring PwDs’ access to nature.
2. The removing barriers program (REB) objective has not been considered in the Canadian legal framework and management guides (C score). In the Spanish case, the law dictates that all barriers should be removed to improve PwDs’ access to parks (A qualification). This mandate has been transposed to the management tools; however, mandatory stipulations do not appear (B qualification).

In Table 4, descriptors for governance rules dimension show:

1. The inclusive legal framework (ILF) applied to Canada clearly demonstrates the lack of provisions in laws and regulations studied (C qualification) as no reference was found on accessibility for PwDs. Despite the fact that no specific regulations or stipulations are in place in the acts, their management tools, such as PCGPOP and Parks Canada Agency Departmental Plan 2019–2020, seem to correct this aspect. The first highlights the idea that the information must be accessible to all visitors, and the second considers accessibility for PwDs as a main goal to be able to foster equitable tourism in parks. No other specific regulations were found (C score) because there is no mention of how to undertake accessibility activities in the park. The analysis for Spain confirms that the legal framework is based on the accessibility statement: inclusive public use of parks is a main objective in the NP act, which is subjected on its ecology integrity. This philosophy has been transposed into the technical documents, so Spain receives an A qualification.
2. Active public participation (APP) evaluated in the Canadian case through laws and regulations shows a good result in terms of participation (A qualification). First Nations and local communities are considered from the start when selecting the potential NP area, so they have the opportunity to be integrated into the whole management decision process. In Spain, public participation is considered in the SNPA. Article 2 promotes collaboration with the public authority and the active participation of individuals or associations. In this line, NP authorities must particularly encourage the involvement of private or public stakeholders. All this is developed further in Articles 35 and 38, with the public participation process and how NP information should be conveyed to citizens. The NP Master Plan also includes a provision on the first point where the park authorities are urged to encourage community collaboration and active participation.
3. Technical accessibility standard (TAS) obtained a C for Canada both within laws and regulations and in guidance and management documents. In the Spanish case, although SNPA indicates accessibility as a requirement in the public use of parks, applying accessibility standards is not required (B score). As the technical documents are inspired by this law, there is once again a lack of information about this point.
4. Outdoor wilderness experience (OWE) is not included in any provisions in Canada’s legal framework for NPs. Only the Parks Canada Agency Departmental Plan 2019–2020 earns a B qualification because accessibility to parks is stipulated as a future improvement. Spanish legal analysis shows no specific provision for WEX, but it is stipulated in the law (B score) because both the SNPA and the network master management plan indicate the importance of promoting accessibility, although without regulating it. A similar situation can be seen in guidelines and management documents: the public use plan does not specify how to achieve an inclusive wilderness experience outdoors.
5. Periodic evaluation program (PEP) is the descriptor most covered in both legal frameworks. The Canadian legal framework earned an A because stewardship reinforces the idea of running periodic evaluations. The Spanish case also includes provisions for periodic evaluations of parks and management plans.

Descriptors for the actors’ dimension as shown in Table 5:

1. Justice equity participation (JEP) is contemplated in the CNPA, point 12(1), with a public consultation requirement to involve citizens and stakeholders, including PwD, in the decision-making process (A qualification). This point develops how the minister responsible for the NP must provide opportunities for public participation at every territorial level, including the participation of aboriginal organizations and representatives from park communities. In guidelines and management documents, this aspect is reinforced in the NP System Plan (1997) (A qualification), although the departmental plan and Yoho NP management plan are not sufficiently specific in terms of inclusiveness for PwDs (B qualification). In Spain, the NP law includes a provision that forces the implementation of park planning instruments to guarantee public participation and accessibility, which is even more fully developed in the NP Master Plan.
2. The park interpreters staff (PIS) descriptor is not considered in any specific regulation (C qualification) in either country. There are no provisions about park staff skills in communication with PwDs in laws or regulations or in guidelines and management documents.

Analysis of the governance resources dimension summarized in Table 6 shows:

1. Inclusive outdoor walkability (IOW) results obtain a C qualification for the Canadian legal framework as none of the provisions have been properly developed. In the case of Spain, there is a similar result for laws and regulations, although the later NP Master Plan states that visitor services must be designed and developed, taking into account universal accessibility. Even so, the study of the park management plans revealed that no specific actions have been developed (B qualification).
2. The website disability information (WDI) indicator obtains a C qualification in both countries as no provisions were identified in the NPs’ legal frameworks on how parks should manage their websites in terms of information for PwDs planning a visit to the park.
3. Educational and informational programs’ (EIP) results do not score better than IOW or WDI in Canadian regulation. There is only one citation in this regard in the Parks Canada Guiding Principles and Operational Policies (PCGPOP). A few more were identified in the Spanish case, resulting in an A and B qualification for the guidance and management documents. These documents develop and highlight the importance of addressing all intersectional realities in the parks’ educational programs.
4. Access to significant natural features (ANA) is not represented in the Canadian legal framework; only the PCGPOP and departmental plan consider this question as a challenge. On the contrary, the Spanish A score for guidance and management documents shows a certain commitment to PwDs’ access to the most significant landscapes in the parks.
5. The transportation systems (TRS) indicator is underdeveloped in Canadian law and regulations, and not specifically for Yoho NP despite its important heritage related to transportation systems. In Spain, legal and regulation items are no better, although management documents received a B score as accessibility is a main objective.

### 3.2. Case Study

Managers of Yoho NP consider inclusiveness to be covered in the NP act because the law indicates that “NPs are dedicated to all Canadians.” However, they recognized that accessibility could be improved with further action. This statement is also valid for the Parks Canada Guiding Principles and Operational Policies and the National Park System Plan. There is, however, no specific framework for including accessibility factors in the management plan actions. The Yoho NP manager admitted that together with reconciliation with First Nations, greening, and sustainability, accessibility will be one of the future core practices. Currently, the NP management plan is being renewed with the intention of including some specifications regarding accessibility. PwD associations, such as the Rocky Mountain Adaptive Association, have been actively involved in the new plan in an effort to take into consideration the realities and opinions of the entire community and add some ideas to incorporate accessibility amenities to the park. In addition, a satisfaction survey was published on the NP’s official website, giving all people the opportunity to participate, but unfortunately, no data for PwDs were collected. The NP manager also commented that the park administration would be interested in exploring new amenities to offer new accessible paths, which can provide new opportunities for people with mobility impairments to explore Yoho landscapes.

Since 1994, the AESM NP has undertaken actions to improve accessibility. This was first done by creating the “Breaking Barriers” program in 1993, and then by building accessibility infrastructure in 1998. These actions were undertaken before the NP management plan was published. AESM NP even received two awards from the Spanish association of people with visual impairments (ONCE). In 2005, a new NP strategic plan was developed, which included new regulations and projects to improve accessibility for PwDs at visitor centers. The park has been in permanent contact with ONCE, looking for new accessibility strategies for the park. Moreover, the Breaking Barriers program undertakes educational actions designed for scholars with special learning needs. According to the manager, the involvement of PwD organizations has benefited the development of the NP management plan. NP managers have data about visitors with disabilities, but it should be noted that people do not always go to the park through the visitor center, making it difficult to fully track them. The NP manager stated in the interview that one of the park’s new objectives would be to develop new paths to create more opportunities for PwDs to explore other landscapes.

In Appendix A and Appendix B, detailed tables for the Yoho (A1) and AESM (A2) NPs list the past and future actions that the park managers have taken and will take concerning accessibility.

## 4. Discussion

We analyzed the governance and legal situation in Canada and Spain through 14 descriptors, applying the framework of policy arrangements structured in dimensions proposed by Liefferink et al. (2006) [39] to answer the main question of how intersectional legal frameworks can contribute to promoting a more inclusive NP for citizens with disabilities.

### 4.1. Dimensions

The discourse dimension shows that principles of equity or fairness are not yet well established in the Canadian system according to results obtained from descriptors’ applicability, in line with Kovacs Bruns and London’s conclusions (2010) [20]. The accessibility topic was not introduced into governance discourse until 2019 when the PCGPOP [61] came into force and in the last two departmental plan reports (2019–2020) [51,52]. In Spain, those principles have been taken into account in its respective legal framework as shown by the nature for all objective (NAO) and removing barriers program (REB) (Table 3), which have been incorporated into the SNPA through the discourse by the allocation of resources and their benefits as a main objective in the act (Art. 5) [60]. Moreover, EUROPARC-España [72] has provided a framework to plan new accessibility plan challenges as a result of an assessment of the Spanish park network using accessibility criteria, reporting the state of the entire park services, indoor and outdoor.

Regarding the NP case studies, the equity and fairness discourse seems to be working on the amendments of national policies and regulations when they are incorporated into the NP plan. Both NPs have been fostering new equity perspectives in guidance and management documents with a clear commitment to favoring access to nature for PwDs. In Canada, the proposal aims to develop guides that provide equal opportunities, integrating informational techniques to make park themes accessible to all visitors. In addition, the Park Plan Management Draft for Yoho expresses a new equity vision in key strategy 2, “True to Place Experiences” (points 4.1 and 4.2) [85]. AESM is pioneering new avenues related to accessibility for PwDs within the park. Its manager aims to explore new options to provide more accessibility paths to points in the park.

Overall, Canada shows a lower level of effectiveness than Spain in terms of rules. In the Canadian legal framework, references to accessibility are missing, when CNPA [49] could have incorporated an intersectional vision to public use of parks, which has been developed in Art. 38 (14.3, c). This is confirmed by a case study of Yoho NP, where insignificant accessibility measures were implemented to improve the wilderness experience through outdoor opportunities. Although in Parks Canada Guiding Principles and Operational Policies (2019) brief references are found on accessibility for PwDs, no technical accessibility standards (TAS) have been included to provide parks amenities like other countries, such as the US and the UK, have [31,70,71,72,85]. Recent management tools, such as the Parks Canada Agency Departmental Plan 2019–2020 and 2020–2021 [55,56], could signify a changing trend.

In the Spanish legal framework, there are specific references to the inclusive public use of national parks (Art. 5) [60]. This approach also influences the NPs’ planning tools, such as the Network Master Plan, where conservation is intersected with inclusive uses, providing them as amenities required by PwDs (Section 1.3-b, Art. 12) [59,84]. Moreover, this park’s legal framework’s effectiveness can be supported by PwD rights regulated beyond just the Spanish Constitution [54], since it has been supplemented with a specific legal PwD framework [21,55]. In addition, as a member of the European Union, Spain must transpose EU directives into its legal system to guarantee equity in active lives for PwDs in society. AESM as a case study seems to be even more effective than the federal rules themselves. The “Breaking Barriers” program, developed in 1993 when the park was designated, has carried out indoor–outdoor accessibility actions, highlighting its pioneering intersectional educational program in line with Sugerman’s [33] suggestions on how to integrate PwDs into outdoor educational activities in parks.

However, neither the Canadian nor the Spanish legal frameworks and technical documents include standard accessibility specifications about how to achieve accessibility communication skills for park staff (PIS) or transportation systems (TRS) for PwDs. Only educational and informational programs (EIP) and access to significant natural features (ANA) show a higher level of effectiveness in Canada and Spain since guidelines and management documents specifically highlight the importance of accessibility referred to in these two items. Specifically, inclusive outdoor wilderness experience (OWE) has been only developed in Spain at the regional–local level through AESM NP public plan uses [84].

The participation principle is guaranteed in terms of rules for stewardship in both countries. Comparative analysis shows several processes for the participation of citizens and stakeholders in decision making either directly or through legitimate intermediaries representing their interests in both countries. However, there is no equitable participation that considers PwDs. In the case of Canada, a good stewardship example is that of First Nations’ involvement in the parks’ decision making [51,52]. However, justice equity participation (JEP) of PwDs is not clearly defined by the park frameworks as it does not regulate which amenities must be included to provide equal participation opportunities for PwDs in those processes [49,53,61]. For instance, Yoho’s management plan renewal is based on a park visitor satisfaction survey, which provides a road map to deal with new park challenges, but no data have been obtained from PwDs, either because there are no visits from this segment of the population or because they did not participate in it. Aware of this lack of information, Yoho’s manager is in touch with a PwD association. In Spain, the extent to which PwD engagement in decision making is made possible is not adequately specified. At the federal level, Law 27/2006, which provided the active public participation framework at the federal level, did not include any representative PwD organizations in its environmental advisory council (Title III, Art. 19) [86]. Locally, AESM has a board of trustees composed of different administrations and entities, which currently does not include any organizations of PwDs, although they might be added in the future. [87].

National park governance accountability seems to have reached similar positive results in both countries. Canada’s NP network has a consistent and well-structured monitoring system through its recurring reporting on park status. Spanish national parks stipulate in SNPA (Art. 21) [60] the parks’ ongoing assessment through the autonomous region’s agency. The Spanish Parks Master Plan mandates an annual statement and a triannual report submitted to the Spanish senate and published publicly [88]. Yoho’s management renovation plan has been successful, and the draft is now on the official park website. Likewise, SNPA states that a use and management master plan, and a public use plan, valid for 10 years, has to be developed after a new NP is sanctioned [60]. When plan revisions take longer than the recommended deadline, the periodic evaluation program descriptor (PEP) should be revised as we found in the AESM management plan dated from 2003 [84]. Disability information on the public website is an essential park qualification tool for people planning visits to NPs [89]. However, the mechanism to provide information through official channels (WDI) [25] has not been addressed in the Canadian or the Spanish NPs’ legal frameworks. Despite the fact that website disability information (WDI) is rarely included in park rules [25], it has been confirmed that the Canadian draft management park plan suggests that WDI is a major challenge. It should provide visitors on the trip cycle with the correct expectations to facilitate their real park experiences [89].

### 4.2. Integrated Analysis of Dimensions

Analysis through the set of proposed dimension descriptors confirms the relevance of developing a specific legal framework to protect PwDs’ rights at the federal level, as stated by Kovacs Bruns and London [20] and Groulx et al. [25]. However, the rights of PwDs to access parks must also be regulated in the NP law itself, considering all intersectional realities in those areas where public use of the park is allowed and regulated, to eliminate barriers and prevent inequalities. This is confirmed by the Spanish parks framework. Even though these rights have been established since 2013 with the latest amended law on PwD rights [55], it does not seem that it has been enough to establish equitable and inclusive outdoor activities in NPs [21].

International treaties offer good guidance for incorporating intersectional realities into NP use, but first, federal legal frameworks and park agencies must integrate them and incorporate them into their governance to address systematic barriers. Canada is on the path to comply with the international treaties on the rights of PwDs, while Spain has 40 years of experience regulating the paradigm of social inclusion [23]. A new promising set of regulations, such as the British Columbia Accessibility Act (2018) [90] and the Accessible Canada Act (ACA) in 2019 [48,91], have provided new opportunities to promote sustainable park tourism, accessible to all able and non-able people [10,25,26,27]. Furthermore, the PCGPOP could incorporate some input from standards published in Canada, such as the recommendation from the BC Building Code [92] or from other countries such as the US or the UK, which have developed accessibility standards for parks [31]. In the Spanish case, there are accessibility standards in other fields, such as those described in the basic document (SUA), specifically chapter 9 on safety of use and accessibility, which could be applied to plan indoor and outdoor infrastructures [93].

Both countries have shown some gaps in parks’ legal frameworks in terms of providing an inclusive environment for PwDs. Good governance is based on cooperative and mutually supportive relationships among all state stakeholders (government, private, and public entities). Moreover, the right to enjoy a full life in society of those sectors of society who have been prevented access to nature and ignored throughout history should not be neglected to comply with ratified international treaties [22,23,24]. This must be taken into consideration as common goals for park agencies to move towards inclusive environments in outdoor park activities in line with sustainable tourism [16,26,27]. In addition, to carry out these types of policies, park rules must be supplemented, incorporating inclusive public park use standards [31]. To meet the needs of PwDs, they must play a participatory role in decision making focused on improving outdoor accessibility activities, contributing to their experience [14,25]. Those decisions should be binding and incorporated into future amendments to park management plans.

### 4.3. Study Limitations

Our analysis of PwDs and NP legal frameworks does not take into account potential additional aspects that could be indirectly linked to the study. We consider that future research should address the information and communication technologies (ICTs) applied to protected natural spaces as a main research topic in both regulation lines and their applicability in a study case. Moreover, to promote an inclusive ICT platform, special mention should be made on both the content and the performance of web-based information to design it to be accessible to all people, incorporating intersectional perspectives. There also exist structural factors of importance, such as environmental, geographical, distance, and population distribution, which can influence NP accessibility. The distance from the most populated areas to the Canadian NPs is much greater than that in Spain, which can be a relevant factor in terms of park visitors.

The objective of analyzing both management documents from two parks is to verify the positive or negative impact of federal level policies on PwDs’ lives and highlight the importance of legislating from the perspective of intersectional realities and needs, since if this is not the case, as has been shown, at lower levels such as the local, the actions carried out may not satisfy the needs of all citizens. Obviously, the NPs chosen are not necessarily representative of other NPs in their countries. Perhaps more case studies could provide a better overview of the park network to evaluate how the application of the legal framework can influence park facilities for each management plan.

In addition, the applicability of parks’ accessibility standards must be adapted to each park’s characteristics. In line with this, future research should undertake intersectional public park use aptitude tests.

## 5. Conclusions

The promotion of inclusive outdoor recreational activities in NPs requires specific policies to promote accessibility for PwDs in federal legislation, which can inspire incorporating inclusiveness input into the regulation of NP laws, where equity should be designated as a main objective.

A comparison of inclusive tools in parks’ legal frameworks in Canada and Spain shows some advances in terms of improving park accessibility. Canada is going to be able to meet the equity challenge in the near future, but it should first consider amending the parks act, emphasizing accessibility in its section on park facilities. Spain has the framework to keep moving forward in equity, but more tools should be added to its parks act.

The dimensions of policy arrangements and FAO principles shed light on the good governance framework. A set of proposed descriptors have offered a holistic way to evaluate the integration of the equity and fairness philosophy into parks’ legal framework. To demonstrate whether there is a framework that has been based on intersectionality, the application of descriptors has started in the field of discourses, then checking that it has permeated into rules, resources, and actors. This has allowed us to verify the status of the issue in both countries.

Improvements based on the descriptors proposed can help to achieve the dimensions of good governance in terms of meeting the principles of effectiveness, participation, and accountability to support sustainable tourism. Consequently, the inclusion of PwDs in park activities implies a standardization of the norms through park accessibility standards at the federal and regional level.

The institutional framework may allow or limit the equity of NP use. Administrations who have the responsibility to correct this lack of equity in the use of parks must endeavor to include PwDs (individuals or through their respective representatives) in participation bodies. For this reason, a report with recommendations will be drawn up for the competent body since the Advisory Council of Environmental Parks (Spain) and the AESM Board of Trustees do not have any PwD association among their members.

Future research should undertake a comprehensive evaluation of information and communication technologies (ICTs) in two research lines: (i) regulations to update legislation in accordance with current social trends and (ii) evaluating content and format from an intersectionality perspective. It is assumed that public use must be inclusive, and it cannot generate park conservation problems. Considering its implementation in the NPs, both NP agencies should undertake to establish an adequate regulatory framework for correct ICT implementation.

## Figures and Tables

**Table 1:** National parks network characteristics in Canada and Spain defined by their respective regulatory frameworks. Canada classifies parks ecology in natural regions between terrestrial (TNR) and marine (MNR); Spain defines it as natural systems (NS). Governance models of NPs have been typified by Dudley (2008) as: (A): governance by government; (B): shared governance; (C): private governance, and (D): governance by indigenous people and local communities.

Dimensions/Concepts	Canada	Spain
National Park Network	47	16
Total land covered by national park protection type in km^2^	450,000.0	3845.9
Percentage land of the country occupied by NP law	2.25	0.76
Percentage land occupied by NPs (national goal) upon completion of the network	3	-
Park network goal according to the ecology classification criteria	39 TNR 29 MNR	40 NS
NP system network achievement as of 2020	28 TNR 5 MNR	12 NS
NP land ownership	100% public (federal government after an official agreement is signed between First Nations and Canadian Government)	82% public (municipality, 45%; State, 20%; and regions, 17%) 18% private
NPs’ governance classification by Dudley (2008)	A (government)	A (government)
NPs’ zoning and uses without accessibility possibilities	Special preservation Wilderness	Reserve Restricted
NPs’ zoning and uses with accessibility possibilities ^1,2^	Natural environment Outdoor recreation Park services	Moderate Special use Traditional settlements

^1^ In Canada, *Natural environment* is the park area where outdoor recreation activities are permitted to raise awareness of the cultural and natural values of the park. *Outdoor recreation* is an area with essential services and facilities whose defining feature is direct access by motorized vehicle. *Park services* is the park area that contains a concentration of visitor services and support facilities [49,50,53]. ^2^ In Spain, *Moderate zone* is the park area where going into the wilderness is permitted and there are low-impact infrastructures because that area combines conservation with cultural values, such as traditional agricultural uses and forestry (communal forest). *Special* is the area where major buildings, facilities, and infrastructures tend to be located within the park if deemed necessary. *Traditional settlements* are an exceptional circumstance where there are populated areas. To ensure citizens’ basic rights, these areas have been established as a one-area land with various uses [59,60].

**Table 2:** Definition and objective of each descriptor to evaluate NPs’ legal framework, related governance dimensions or policy practices in the tetrahedron model proposed by Liefferink et al. (2006) [33], and some of the FAO key principles (2011) [32].

Dimension	Descriptor ^1^	Definition	Principle
Discourses	Nature for all objective (NAO)	The extent to which universal accessibility is included as a planning objective.	Equity/fairness
	Removing barriers program (REB)	The extent to which detection, elimination, and preventing barriers is included as a planning objective.	
Rules	Inclusive legal framework (ILF)	The inclusion of accessibility standards in the federal regulatory frameworks.	Effectiveness
	Active public participation (APP)	An intersectional and active institutional participation in policymaking with powers and responsibilities defined by NPs’ legal framework.	Participation
	Technical accessibility standard (TAS)	The inclusion of accessibility standards in NP management plan.	Effectiveness
	Outdoor wilderness experience (OWE)	Active involvement in outdoor activities.	
	Periodic evaluation program (PEP)	Periodic evaluations to detect issues in accessibility services.	Accountability
Actors	Justice equity participation (JEP)	The extent to which the legal framework provides opportunities to minorities to become involved in the decision-making process voluntarily or mandatorily.	Participation
	Park interpreter staff (PIS)	Staff is required to have accessibility skills or provided with training to improve theirs.	Effectiveness
Resources	Inclusive outdoor walkability (IOW)	Amenities specified by law to improve outdoor accessibility.	Effectiveness
	Website disability information (WDI)	The extent to which outdoor accessibility is specified on park’s official digital platforms.	Accountability
	Educational and informational programs (EIP)	Educational programs based on inclusiveness.	Effectiveness
	Access to significant natural features (ANA)	Access for all people to significant natural and cultural park points.	Effectiveness
	Transportation systems (TRS)	Accessible land and water transport within the parks’ perimeters.	Effectiveness

^1^ Descriptors have been defined to closely assess the legal framework of national parks. Each descriptor is designed to study all relevant aspects for full inclusion of PwMDs.

**Table 3:** Comparison table of the governance discourse dimension for the nature for all (NAO) and removing barriers program (REB) objectives.

Descriptor	NAO	REB
FAO Principle	Equity/Fairness	Equity/Fairness
**Canada: Law and regulation ^1^**		
Canada National Parks Act (CNPA)	C	C
Canada National Marine Conservation Areas Act	C	C
Parks Canada Agency Act	NA	NA
Historic Sites and Monuments Act	NA	C
NPs’ General Regulations	C	C
Heritage Railway Stations Protection Act	NA	C
**Canada: Guidelines and management documents ^1^**		
Parks Canada Guiding Principles and Operational Policies (PCGPOP)	B	C
NP System Plan (1997)	C	C
Parks Canada Agency Departmental Plan 2019–2020	A	C
Yoho NP of Canada Management Plan (2010)	C	C
**Spain: Law and regulation ^1^**		
Spanish National Parks Act (SNPA)	A	A
Natural and Biodiversity Heritage Law	NA	NA
AESM national park creation decree, 21 October 1955	C	C
Law 7/1988 of AESM NP reclassification	NA	NA
**Spain: Guidelines and management documents ^1^**		
NP Master Plan	A	B
Uses and Management Master Plan	A	B
AESM NP Public Use Plan	A	B

^1^ Typified scale: A: provisions in the law; B: no specific provision but stipulated in the law; C: no specific regulation; NA: not applicable, because not focused on the wilderness experience.

**Table 4:** Comparison table for the governance rules dimension, focusing on inclusive legal framework (ILF), active public participation (APP), technical accessibility standard (TAS), outdoor wilderness experience (WEX), and periodic evaluation program (PEP).

Descriptor	ILF	APP	TAS	OWE	PEP
FAO Principle	Effectiveness	Participation	Effectiveness	Effectiveness	Accountability
**Canada: Law and regulation ^1^**					
Canada National Parks Act (CNPA)	C	A	C	C	A
Canada National Marine Conservation Areas Act	C	A	C	C	A
Parks Canada Agency Act	NA	NA	NA	NA	A
Historic Sites and Monuments Act	C	NA	C	NA	A
NP General Regulations	C	NA	C	C	C
Heritage Railway Stations Protection Act	C	NA	C	NA	C
**Canada: Guidelines and management documents ^1^**					
Parks Canada Guiding Principles and Operational Policies (PCGPOP)	B	A	C	B	A
NP System Plan (1997)	NA	A	NA	NA	A
Parks Canada Agency Departmental Plan 2019–2020	A	B	C	B	A
Yoho NP of Canada Management Plan (2010)	C	C	C	C	A
**Spain: Law and regulation ^1^**					
Spanish National Parks Act (SNPA)	A	A	B	B	A
Natural and Biodiversity Heritage Law	B	A	NA	NA	A
AESM national park creation decree	C	C	C	C	C
Law 7/1988 of AESM NP reclassification	NA	C	NA	NA	NA
**Spain: Guidelines and management documents ^1^**					
NP Master Plan	A	A	B	B	A
Uses and Management Master Plan	A	A	C	B	A
AESM NP Public Use Plan	A	A	B	A	A

^1^ Typified scale: A: provisions in the law; B: no specific provision but stipulated in the law; C: no specific regulation; NA: not applicable, because not focused on the wilderness experience.

**Table 5:** Comparison table for the actor dimension in relation to justice equity participation (JEP) and park interpreters staff (PIS).

Descriptors	JEP	PIS
FAO Principle	Participation	Effectiveness
**Canada: Law and regulation ^1^**		
Canada National Parks Act (CNPA)	A	C
Canada National Marine Conservation Areas Act	C	C
Parks Canada Agency Act	NA	B
Historic Sites and Monuments Act	NA	C
NP General Regulations	NA	NA
Heritage Railway Stations Protection Act	C	C
**Canada: Guidelines and management documents ^1^**		
Parks Canada Guiding Principles and Operational Policies (PCGPOP)	B	C
NP System Plan (1997)	A	NA
Parks Canada Agency Departmental Plan 2019–2020	B	C
Yoho NP of Canada Management Plan (2010)	B	C
**Spain: Law and regulation ^1^**		
Spanish National Parks Act (SNPA)	A	C
Natural and Biodiversity Heritage Law	A	NA
AESM national park creation decree	C	C
Law 7/1988 of AESM NP reclassification	NA	NA
**Spain: Guidelines and management documents ^1^**		
NP Master Plan	A	C
Uses and Management Master Plan	A	C
AESM NP Public Use Plan	A	C

^1^ Typified scale: provisions in the law; B: no specific provisions but stipulated in the law; C: no specific regulation; NA: not applicable, because not focused on wilderness experience.

**Table 6:** Comparison table for the resources dimension in relation to inclusive outdoor walkability (IOW), website disability information (WDI), educational and informational programs (EIP), access to significant natural features (ANA), and transportation systems (TRS).

Descriptor	IOW	WDI	EIP	ANA	TRS	
FAO Principle	Effectiveness	Accountability	Effectiveness	Effectiveness	Effectiveness	
**Canada: Law and regulation ^1^**						
Canada National Parks Act (CNPA)	C	C	C	C	C	
Canada National Marine Conservation Areas Act	C	C	C	C	C	
Parks Canada Agency Act	C	C	C	C	C	
Historic Sites and Monuments Act	C	C	C	C	C	
NP General Regulations	C	C	C	C	C	
Heritage Railway Stations Protection Act	NA	C	C	NA	C	
**Canada: Guidelines and management documents ^1^**						
Parks Canada Guiding Principles and Operational Policies (PCGPOP)	C	C	B	B	C	
NP System Plan (1997)	NA	NA	C	NA	NA	
Parks Canada Agency Departmental Plan 2019–2020	C	C	C	B	C	
Yoho NP of Canada Management Plan (2010)	C	C	C	C	C	
**Spain: Law and regulation ^1^**						
Spanish National Parks Act (SNPA)	C	C	C	B	C	
Natural and Biodiversity Heritage Law	NA	NA	C	NA	NA	
AESM NP creation decree	C	C	C	C	C	
AESM NP reclassification law	C	C	C	C	C	
**Spain: Guidelines and management documents ^1^**						
NP Master Plan	A	C	B	A	C	
Uses and Management Master Plan	B	C	A	A	B	
NP Public Use Plan	B	C	A	A	B	

^1^ Typified scale: A: provisions in the law; B: no specific provisions but stipulated in the law; C: no specific regulation; NA: not applicable, because of missing to focus on wilderness experience.

## Data Availability

The data will be made available on request from the corresponding author.

## Appendix A. Yoho National Park Questionnaire (Interview Was Undertaken in a Local Language, English)

1. Is inclusive use reflected in the park’s governance, legislative frameworks, and management plans? If so, please explain.
  - National Parks Act (1979). Last amended on 12 December 2017. Could you briefly explain your response?
    ◦ Yes
    ◦ No
  - National Parks General Regulations (SOR/78-213) last amended on 23 November 2018. Could you briefly explain your response?
    ◦ Yes
    ◦ No
  - National Park System Plan (1997). Could you briefly explain your response?
    ◦ Yes
    ◦ No
  - Parks Canada Guiding Principles and Operational Policies. Could you briefly explain your response?
    ◦ Yes
    ◦ No
  - Park-specific management plan (Yoho National Park, 2010). Could you briefly explain your response?
    ◦ Yes
    ◦ No

When responding to questions 2, 3, and 4, please use Table A1.

2. Which, if any, accessibility improvements have you observed since your park’s management plan was approved in 2010?
3. Have there been investments in accessible infrastructure since the management plan was approved? If so, using Table A1 below, indicate where the areas and programs highlighted in the 2010 management plan are.
4. In your opinion, has the implementation of accessibility improvements been successful in promoting universal use? In what ways? What do you think could be improved?

**Table A1:** Yoho National Park data table results based on the accessibility actions highlighted in the 2010 management plan.

Management Plan Actions	Accessibility Improvement	Investment or Budget	Funding Type (Pub/Private)	Funding’s Organization	Start Date	Finish Date	Outcome	Challenges
**Hiking programs, park exhibits, and electronic media**	Interpretive panels in the town field with braille for visually impaired	Unknown, project is +12 years old	Government	Parks Canada			Positive	Keeping offer updated and ensuring products met the needs of user groups
**Reception and orientation at both west and east entrances**	Accessible washrooms at east entrance	CAD 360,000	Government	Parks Canada	2013	2017	Positive	
**Expand range of recreational, leisure, and learning opportunities**	None							
**Infrastructure at Kicking Horse and Monarch Campgrounds**	New in 2020, accessible washroom and shower building	CAD 1,615,000		Parks Canada	2017	2019	Positive	
**Winter recreation opportunities**	None							
**Kicking Horse Pass to the Last Spike Cultural Landscape**		Unknown		Parks Canada				
**Target youth, urban Canadians, and new Canadians: Kicking Horse Pass, the Spiral Tunnels, and the Burgess**	None							
**Shale materials to communicate the significance of cultural resources and the World Heritage Site**	Accessible digital interpretation for remote access	CAD 1000		Parks Canada	2020	2020	Positive	Satellite/cell phone reception, reaching broader audiences
**Special events and new recreational activities that promote public understanding and appreciation of the park**	None			Parks Canada				

5. Have you worked with associations or groups that represent the interests of people with mobility impairments when making the improvements outlined above?
6. Are you monitoring park visits? If so, are visitors registered in terms of visitor profile, and specifically, do you keep track of visitors with mobility impairments? Can you share those visitor statistics with us? Do you have any other information available on park use by visitors with mobility impairments?
7. Finally, as an expert on the Yoho National Park, would you be interested in exploring the possibility of assessing and designing new accessible trails to facilitate access to the NP’s landscapes/resources for wheelchair users?

Questions 8 and 9 only refer to the Yoho National Park when park management is actually involved in a renewal process (table is available to help you answer the following questions)

8. The new park management plan is in the process of being renewed; which associations are participating in this development? Are those data available?
9. In your state of the park assessment (SOPA), you concluded that the results have been “good” in increasing the number of park visitors and their enjoyment of the park. The next question is to know if you included visitors with disability (and how many of them there were) in this satisfaction survey. Do you believe that this evaluation should include people with disability to produce a realistic map of society?

## Appendix B. Aigüestortes i Estany de Sant Maurici National Park Questionnaire (Interview was Undertaken in a Local Language, Spanish)

1. As NP director, could you please provide your perspective, interpretation, and opinion regarding compliance with the accessibility objectives of the following documents:
  - Law 30/2014. Could you briefly explain your answer?
    ◦ Yes
    ◦ No
  - Decree 39/2003 of 4 February approving the NP’s Guiding Use and Management Plan (PRUG). Could you briefly explain your answer?
    ◦ Yes
    ◦ No
  - Public Use Plan (PUP). Could you briefly explain your answer?
    ◦ Yes
    ◦ No

Please use Table A2 when answering the following questions about the actions listed in Article 12 of the Public Use Plan.

2. What improvements in terms of accessibility have been made since the approval of the national park’s PRUG and PUP? Could you provide current data on what kinds of improvements have been made in the following items (PUP Article 12)?
3. Have there been any investments in infrastructure and equipment to improve accessibility since the PRUG (2003) and the PUP were approved? Could you provide current data on investments made in the following items (PUP Article 12)?
4. In your opinion, has the implementation of accessibility improvements been successful in promoting inclusive use of the national park? How have you seen that effect to be positive or negative from a social and environmental point of view?

**Table A2:** Aigüestortes i Estany de Sant Maurici data table results based on the accessibility actions highlighted in the 2010 management plan.

PUP Actions	Accessibility Improvement	Investment and/or Budget	Funding Type (Public/Private)	Funding Organization	Start Date	Finish Date	Outcome (Positive and Negative)	Challenges
**Education and** **environmental interpretation programs**	“Breaking Barriers” program: specifically dedicated to people with disabilities	EUR 12,000/year + NPs workers	Public	NP authority and Regional Government of Catalonia	1993	-	Positive: 2000 students/year, 1000 of them local, as well as around 2000 visitors enjoyed the environmental educational activities. Additionally, activities organized with the ASPID foundation, which are focused on special needs’ educational centers.	To promote guided visitations. To provide visitors and give students opportunities to visit villages inside the park area. Accessibility program evaluation with ONCE. Searching for new funding to develop a new park accessibility plan.
**Enjoyment** **of nature:** **new options** **offered**	Accessibility materials (tactile/transportable models)	±EUR 5000/year +NP personal staff	Public	NP authority and Catalonia Administration	2005	-	Positive: Huge diversity of indoor and outdoor nature activities (e.g., Starlight).	Deseasonalize tourism with a more well-rounded offering of activities.
**Visitor** **centers**	Periodic accessibility evaluation of visitor centers to improve it	EUR 50,000/year	Public	NP authority and Catalonia Administration		-	Positive: Visitor centers receive and inform visitors and have free Wi-Fi, permanent exhibitions, and audiovisual materials in many languages.	Adapt/promote heritage buildings, such as visitor centers, especially those located at the main entrances.
**Paths**	There are 6 paths with available length to people with disabilities	Since 1993, the park has made paths more accessible. In 2008. Obra Social la Caixa funded building two accessible catwalks in the park	Public	NP Catalonia and local Administrations	1993	-	Positive: Acceptable pathway network (200 km), 8–10 km available to people with disabilities located in 6 different and significant landscapes, including high mountains.	Strike a balance between high mountains and visitor security. Be able to provide updated information on the trails (especially in winter). Analyzing new proposals to expand accessible path network.
**Other** **services and/or** **public use equipment**	Access to the parking lot at the “San Mauricio” lake and the “Planell” area through standard disability permits		Public	NP authority and Catalonia Administration		-	Positive: Carros de Foc path tourism program, all mountain shelters, along the path, work together promoting local products. Los Pastores ecomuseum with livestock activity promotion. Toirigo Environment Informational Center and Campsite. Sale of informational material at visitor centers. Negative: The park has detected that some people park their cars in disabled parking-only spots. Unfortunately, the park cannot punish infractors because it does not have the power to do so.	
**Signage**	Signs in braille	EUR 10,000/year	Public	NP authority, Catalonia and local administrations	2000	-	Positive: Good signage even in remote and high mountain areas. Billboards and maps in the main park entrances and villages. Negative: Problem with signage maintenance in winter.	Improvement of signage system taking into consideration distances and unevenness. Currently only time it takes is indicated.
**Public** **transport**	Not available		Public	Catalonia Administration		-	Positive: Access by taxi available to most emblematic park points. In summer, visitors can travel by cable car located in the south or take a bus around the park. Only available in summer.	Ensure entire loop of bus all year round (or most of the year).

5. When implementing accessibility improvements and, at the time, drawing up the PUP, was the opinion/collaboration of people, associations, and/or representatives that defend the interests of persons with disabilities taken into account? If so, could you please name the organizations that participated in the process?
6. Is there a record of visits to the national park? If so, are visitors being registered with additional personal information, including a record of visitors with disabilities using any sort of classification? If so, could you please share with our research team the statistical data? Do you have any other information about visitors with reduced mobility?
7. Finally, and knowing that there are already some accessible routes, we would like to know from your technical perspective and as an expert on the natural resources of the national park if you think it would be feasible to explore new entrances and routes to expand the network in terms of accessibility and to offer new landscapes/natural resources in the park to people with motor disabilities.

## References

[1] Lackey N.Q., Tysor D.A., McNay G.D., Joyner L., Baker K.H., Hodge C. (2019). Mental health benefits of nature-based recreation: A systematic review. Ann. Leis. Res..

[2] Zhang G., Poulsen D.V., Lygum V.L., Corazon S.S., Gramkow M.C., Stigsdotter U.K. (2017). Health-promoting nature access for people with mobility impairments: A systematic review. Int. J. Environ. Res. Public Health.

[3] Siikamäki P., Kangas K., Paasivaara A. (2015). Biodiversity attracts visitors to national parks. Biodivers. Conserv..

[4] Bertzky B., Corrigan C., Kemsey J., Kenney S., Ravilious C., Besançon C., Burgess N. (2012). Protected Planet Report 2012: Tracking Progress towards Global Targets for Protected Areas (2012).

[5] Dudley N. (2013). Guidelines for Applying Protected Area Management Categories.

[6] Lausche B. (2011). Guidelines for Protected Areas Legislation.

[7] Thede A.K., Haider W., Rutherford M.B. (2014). Zoning in national parks: Are Canadian zoning practices outdated?. J. Sustain. Tour..

[8] Leung Y.-F., Spenceley A., Hvenegaard G., Buckley R. (2018). Tourism and Visitor Management in Protected Areas: Guidelines for Sustainability. Best Practice Protected Area Guidelines.

[9] Muñoz L., Hausner V.H. (2013). What do the IUCN categories really protect? A case study of the alpine regions in Spain. Sustainability.

[10] Pérez-Calderón E., Prieto-Ballester J.M., Miguel-Barrado V., Milanés-Montero P. (2020). Perception of sustainability of Spanish national parks: Public use, tourism and rural development. Sustainability.

[11] Devine M.A. (2004). “Being a ‘doer’ instead of a ‘viewer’”: The role of inclusive leisure contexts in determining social acceptance for people with disabilities. J. Leis. Res..

[12] Shen X.J., Huang R.H., Zhang J.S. (2019). U.S. national parks accessibility and visitation. J. Mt. Sci..

[13] Corazon S.S., Christoffersen Gramkow M., Varning Poulsen D., Linn Lygum V., Zhang G., Karlsson Stigsdotter U. (2019). I would really like to visit the forest, but it is just too difficult: A qualitative study on mobility disability and green spaces. Scand. J. Disabil. Res..

[14] Farrington J.H. (2007). The new narrative of accessibility: Its potential contribution to discourses in (transport) geography. J. Transp. Geogr..

[15] Setola N., Marzi L., Torricelli C. (2018). Accessibility indicator for a trails network in a Nature Park as part of the environmental assessment framework. Environ. Impact Assess. Rev..

[16] Bianchi P., Cappelletti G.M., Mafrolla E., Edgardo Sica E., Sisto R. (2020). Accessible tourism in natural park areas: A social network analysis to discard barriers and provide information for people with disabilities. Sustainability.

[17] Iwarsson S., Sthal A. (2003). Accessibility, usability and universal design—Positioning and definition of concepts describing person-environment relationships. Disabil. Rehabil..

[18] United Nations Universal Declaration of Human Rights 1948. https://www.un.org/en/about-us/universal-declaration-of-human-rights.

[19] United Nations Department of Economic and Social Affairs Disability. The Standard Rules on the Equalization of Opportunities for Persons with Disabilities (A/RES/48/96)..

[20] Kovacs Bruns K., London G.L. (2010). Analyzing the impact of disability legislation in Canada and the United States. J. Disabil. Policy Stud..

[21] Citarella A., Sanchez Iglesias A.I., González Ballester S., Gentil Gutiérrez A.N., Maldonado Briegas J.J., Vicente Castro F., Gozález Bernal J. (2020). Being disabled persons in Spain: Policies, stakeholders and services. Int. J. Dev. Educ. Psychol. INFAD Rev. Psicol..

[22] Lombardo E., Verloo M. (2009). Institutionalizing Intersectionality in the European Union?. Int. Fem. J. Polit..

[23] Watson B., Scraton S.J. (2013). Leisure studies and intersectionality. Leis. Stud..

[24] Mumbi Maina-Okori N., Renee Koushik J., Wilson A. (2018). Reimagining intersectionality in environmental and sustainability education: A critical literature review. J. Environ. Educ..

[25] Groulx M., Lemieux C., Freeman S., Cameron J., Wright P.A., Healy T. (2021). Participatory planning for the future of accessible nature. Local Environ..

[26] Sisto R., Cappelletti G.M., Bianchi P., Sica E. (2021). Sustainable and accessible tourism in natural areas: A participatory approach. Curr. Issues Tour..

[27] Sica E., Sisto R., Bianchi P., Giulio Cappelletti G. (2021). Inclusivity and responsible tourism: Designing a trademark for a national park area. Sustainability.

[28] Wirtz Z., Hagerman S., Hauer R.J., Konijnendijk van den Bosch C. (2021). What makes urban forest governance successful?—A study among Canadian experts. Urban For. Urban Green..

[29] Perry M., Cotes L., Horton B., Kunac R., Snell I., Taylor B., Wright A., Devan H. (2021). Enticing” but not necessarily a “space designed for me”: Experiences of urban park use by older adults with disability. Int. J. Environ. Res. Public Health.

[30] Lawrence A., De Vreese R., Johnston M., Konijnendijk van den Bosch C., Sanesi G. (2013). Urban forest governance: Towards a framework for comparing approaches. Urban For. Urban Green..

[31] Newman & Park (n/i) Accessibility Standards in Outdoor Environments. https://citeseerx.ist.psu.edu/viewdoc/download?doi=10.1.1.626.4095&rep=rep1&type=pdf.

[32] Menzies A., Mazan C., Borisoff J.F., Mattie J.L., Ben Mortenson W.B. (2021). Outdoor recreation among wheeled mobility users: Perceived barriers and facilitators. Disabil. Rehabil. Assist. Technol..

[33] Sugerman D. (2001). Inclusive outdoor education: Facilitating groups that include people with disabilities. J. Exp. Educ. Winter.

[34] Pinna F., Garau C., Annunziata A., Gervasi O. (2021). A literature review on urban usability and accessibility to investigate the related criteria for equality in the city. Computational Science and Its Applications—ICCSA 2021.

[35] Arts B., Leroy P. (2006). Institutional Dynamics in Environmental Governance.

[36] FAO (2011). Framework for Assessing and Monitoring Forest Governance. The Program on Forests Food and Agriculture Organization (PROFOR).

[37] Campese J., Nakangu B., Silverman A., Springer J. IUCN Commission on Environmental, Economic and Social Policy 2016. Natural Resource Governance Framework Assessment Guide. https://www.iucn.org/commissions/commission-environmental-economic-and-social-policy/our-work/knowledge-baskets/natural-resource-governance.

[38] Convection on Biological Diversity (1992). List of the Parties. https://www.cbd.int/information/parties.shtml.

[39] Liefferink D., Arts B., Leroy P. (2006). The dynamics of policy arrangements: Turning round the tetrahedron. Institutional Dynamics in Environmental Governance.

[40] Creswell J.W. (2018). Qualitative Inquiry & Research Design: Choosing Among Five Approaches.

[41] Government of Canada The Government of Canada Tables the Optional Protocol to the United Nations Convention on the Rights of Persons with Disabilities. https://www.canada.ca/en/employment-social-development/news/2017/11/the_government_ofcanadatablestheoptionalprotocoltotheunitednatio.html.

[42] Government of Canada (1993). Ratification of the Convention on Biological Diversity (CBD). https://www.canada.ca/en/environment-climate-change/corporate/international-affairs/partnerships-organizations/biological-diversity-convention.html.

[43] Gobierno de España Ministerio de la Presidencia, Relación con las Cortes y Memoria Democrática. Agencia Estatal Boletín Oficial del Estado. Instrumento de Ratificación de la Convección sobre los Derechos de las Personas con Discapacidad, Hecho en Nueva York el 13 de Diciembre de 2006. BOE» núm. 96, de 21 de Abril de 2008, Páginas 20648 a 20659 (12p). https://www.boe.es/eli/es/ai/2006/12/13/(1).

[44] Gobierno de España (1992). Ministerio para la transición ecológica y reto demográfico. Convenio sobre la Diversidad Biológica. https://www.miteco.gob.es/es/biodiversidad/temas/conservacion-de-la-biodiversidad/conservacion-de-la-biodiversidad-en-el-mundo/cb_mundo_convenio_diversidad_biologica.aspx.

[45] López H. (2014). Derecho Romano y el Sistema Jurídico Anglosajón. https://derechoromanounivia.wordpress.com/2014/08/07/el-derecho-romano-y-el-sistema-juridico-anglosajon/.

[46] Government of Canada (1981). Canadian Charter of Rights and Freedoms. https://open.canada.ca/data/en/dataset/06d31e10-a2a8-4d53-9ff3-567714a0a9f3.

[47] Justice Laws Website Canadian Human Rights Act (R.S.C., 1985, c. H-6). https://laws.justice.gc.ca/eng/acts/H-6/rpdc.html.

[48] Jacobs L., Anderson M., Rohr R., Perry T. (2021). The Annotated Accessible Canada Act—Complete Text. https://scholar.uwindsor.ca/lawpub/12.

[49] Government of Canada Justice Laws Website. Canada National Parks Act (S.C. 2000, c. 32). https://laws-lois.justice.gc.ca/eng/acts/N-14.01/.

[50] Needham M.D., Dearden P., Rollins R., McNamee K. (2016). Parks and Protected Areas in Canada. Planning and Management.

[51] Government of Canada (2019). Parks Canada. 2019–20 Departmental Plan. https://www.pc.gc.ca/en/agence-agency/bib-lib/plans/dp/dp2019-20/index.

[52] Government of Canada (2020). Parks Canada. 2020–21 Departmental Plan. https://www.pc.gc.ca/en/agence-agency/bib-lib/plans/dp/dp2020-21/index.

[53] Government of Canada Parks Canada. National Park System Plan. Canadian Heritage Parks Canada, Canada, 1997; pp. 2–13, 20–21. http://parkscanadahistory.com/publications/system-plan-eng-1.pdf.

[54] Gobierno de España Ministerio de la Presidencia, Relación con las Cortes y Memoria Democrática. Agencia Estatal Boletín Oficial del Estado. Constitución Española, 1978. BOE» núm. 311, de 29 December 1978. https://www.boe.es/eli/es/c/1978/12/27/(1)/con.

[55] Gobierno de España Ministerio de la Presidencia. Relación con las Cortes y Memoria Democrática. Agencia Estatal Boletín Oficial del Estado. Real Decreto Legislativo 1/2013, de 29 de Noviembre, por el que se Aprueba el Texto Refundido de la Ley General de las Personas con Discapacidad y de su Inclusión Social. BOE» núm. 289, de 3 de Diciembre de 2013, Páginas 95635 a 95673 (39p). https://www.boe.es/eli/es/rdlg/2013/11/29/1.

[56] Santamarina Campos B. (2019). El inicio de la protección de la naturaleza en España. Orígenes y balance de la conservación. Rev. Española Investig. Sociol..

[57] Almenar-Muñoz M. (2016). El reciente avance en la protección de las zonas húmedas en la Comunidad Valenciana. Actual. Jurídica Ambiental..

[58] López-Ramón F. (2014). Trayectoria del régimen jurídico de los Parques Nacional en España. Ambient. Rev. Minist. Medio Ambiente.

[59] Gobierno de España Ministerio de la Presidencia, Relación con las Cortes y Memoria Democrática. Agencia Estatal Boletín Oficial del Estado. Real Decreto 389/2016 de 22 de Octubre por el que se Aprueba el Plan Director de la Red de Parques Nacionales. «BOE» núm. 257, de 24 de Octubre de 2016, Páginas 74051 a 74076 (26p). Departamento: Ministerio de Agricultura, Alimentación y Medio Ambiente. Ref.: BOE-A-2016-9690. https://www.boe.es/eli/es/rd/2016/10/22/389.

[60] Gobierno de España Ministerio de la Presidencia, Relación con las Cortes y Memoria Democrática. Agencia Estatal Boletín Oficial del Estado. Ley 30/2014 de 3 de Diciembre de Parques Nacionales. BOE» núm. 293, de 4 de Diciembre de 2014, Páginas 99762 a 99792 (31 págs.). https://www.boe.es/eli/es/l/2014/12/03/30.

[61] Government of Canada (2018). Parks Canada. Parks Canada Guiding Principles and Operational Policies. https://www.pc.gc.ca/en/docs/pc/poli/princip.

[62] Government of Canada Yoho National Park. Nature, Science and Culture. https://www.pc.gc.ca/en/pn-np/bc/yoho/culture.

[63] Government of Canada The Recognition and Reconciliation of Rights Policy for Treaty Negotiations in British Columbia. https://www.rcaanc-cirnac.gc.ca/eng/1567636002269/1567636037453.

[64] Gobierno de España Ministerio Para la Transición Ecológica y reto Demográfico. Aigüestortes i Estany de Sant Maurici: Valores Culturales. https://www.miteco.gob.es/es/red-parques-nacionales/nuestros-parques/aiguestortes/valores-naturales/default.aspx.

[65] Statista Research Department (2021). National Park Visitors in Canada 2011–2020. https://www.statista.com/statistics/501269/national-park-visitors-in-canada/#:~:text=National%20park%20visitors%20in%20Canada%202011%2D2020&text=In%20the%20fiscal%20year%202019,15.9%20million%20the%20previous%20year.&text=The%20national%20parks%20of%20Canada%20are%20managed%20by%20Parks%20Canada%20Agency..

[66] Gobierno de España Ministerio para la Transición Ecológica y el Reto Demográfico. Red de Parques Nacionales: Visitantes. https://www.miteco.gob.es/es/red-parques-nacionales/la-red/gestion/visitasppnn_tcm30-67283.pdf.

[67] Worrell R., Appleby M.C. (1999). Stewardship of natural resources: Definition, ethical and practical aspects. J. Agric. Environ. Ethics.

[68] Saner M., Wilson J. Stewardship, Good Governance and Ethics (1 December 2003). Institute On Governance Policy Brief No. 19. https://papers.ssrn.com/sol3/papers.cfm?abstract_id=1555815.

[69] EUR-Lex Access to European Union Law. https://eur-lex.europa.eu/homepage.html?locale=en.

[70] United States Department of the Interior (2000). National Park Service. Director’s Order No. 42: Accessibility for Visitors with Disabilities. https://www.nps.gov/subjects/policy/upload/DO_42_11-3-2000.pdf.

[71] Fieldfare Trust (2005). Countryside for All Good Practice Guide. A Guide to Disabled People’s Access in the Countryside.

[72] EUROPARC-España (2005). Manual Sobre Conceptos de uso Público en los Espacios Naturales Protegidos.

[73] EUROPARC-España (2007). Catálogo de Buenas Prácticas en Materia de Accesibilidad en Espacios Naturales Protegidos.

[74] U.S. Department of the Interior (2006). National Park Service. Management Policies. The Guide to Managing the National Park System. https://www.nps.gov/policy/mp/policies.html#_Toc157232893.

[75] Government of Canada Justice Laws Website. Canada National Marine Conservation Areas Act (S.C.2002, c. 18). https://laws-lois.justice.gc.ca/eng/acts/c-7.3/.

[76] Government of Canada Justice Laws Website. Parks Canada Agency Act (S.C. 1998, c.31). https://laws-lois.justice.gc.ca/eng/acts/P-0.4/.

[77] Government of Canada Justice Law Website. Historic Sites and Monuments Act, R.S.C., 1985, c. H-4 (2013). https://laws-lois.justice.gc.ca/eng/acts/H-4/page-1.html.

[78] Government of Canada Justice Laws Website. National Parks General Regulations (SOR/78-213). https://laws-lois.justice.gc.ca/eng/regulations/sor-78-213/index.html.

[79] Government of Canada Justice Laws Website. Heritage Railway Stations Protection Act, R.S.C.1985, c.52 (4th Supp.). https://laws-lois.justice.gc.ca/eng/acts/h-3.5/.

[80] Government of Canada (2010). Parks Canada. Yoho National Park. Park Management Plan. https://www.pc.gc.ca/en/pn-np/bc/yoho/gestion-mgmt/plandirecteur-mgmtplan.

[81] Gobierno de España Ministerio de la Presidencia, Relaciones con las Cortes y Memoria Democrática. Agencia Estatal del Boletín Oficial del Estado. Ley 42/2007, de 13 de Diciembre, del Patrimonio Natural y de la Biodiversidad. https://www.boe.es/eli/es/l/2007/12/13/42/con.

[82] Gobierno de España Ministerio de la Presidencia, Relaciones con las Cortes y Memoria Democrática. Agencia Estatal del Boletín Oficial del Estado. Ley 7/1988, de 30 de Marzo, de Reclasificación del Parque Nacional de Aigüestortes y Lago de Sant Maurici. https://www.boe.es/eli/es-ct/l/1988/03/30/7.

[83] Gobierno de España Ministerio para la Transición Ecológica y Reto Demográfico. Decreto 39/2003 de 4 de Febrero de 2003 del Plan Rector de Uso y Gestión de Aigúestortes i l´Estany de Sant Maurici. DOGC nº 3825. https://www.miteco.gob.es/es/red-parques-nacionales/la-red/gestion/prug_aigues_tcm30-59508.pdf.

[84] Gencat Parcs Naturals de Catalunya. Plà d´Us Públic del Parc Nacional de Aigüestortes I Estany de Sant Maurici. http://parcsnaturals.gencat.cat/web/.content/Xarxa-de-parcs/aiguestortes/Coneix-nostra-feina/instruments-planificacio/plans-programes-especifics/pla-us-public/PDF/43_109693.pdf.

[85] Government of Canada (2021). Parks Canada. Yoho National Park of Canada Draft Management Plan. https://www.pc.gc.ca/en/pn-np/bc/yoho/gestion-mgmt/plandirecteur-mgmtplan/ebauche-draft.

[86] Gobierno de España Ministerio de la Presidencia, Relaciones con las Cortes y Memoria Democrática. Ley 27/2006, de 18 de Julio, por la que se Regulan los Derechos de Acceso a la Información, de Participación Pública y de Acceso a la Justicia en Materia de Medio Ambiente (Incorpora las Directivas 2003/4/CE y 2003/35/CE). BOE» núm. 171, de 19/07/2006. https://www.boe.es/eli/es/l/2006/07/18/27/con.

[87] Gencat Parc Nacionla d´Aigüestortes i Estany de Sant Maurici. Òrgan Rector. http://parcsnaturals.gencat.cat/ca/xarxa-de-parcs/aiguestortes/coneix-la-nostra-feina/qui-som/organ-rector/.

[88] Gobierno de España Vicepresidencia Tercera del Gobierno. Ministerio para la Transición Ecológica y el Reto Demográfico. Plan de Seguimiento y Evaluación de la Red de Parques Nacionales, 2016; p. 72. https://www.miteco.gob.es/es/red-parques-nacionales/plan-seguimiento-evaluacion/seguimiento.aspx.

[89] Buhalis D., Michopoulou E. (2011). Information-enabled tourism destination marketing: Addressing the accessibility market. Curr. Issues Tour..

[90] British Columbia Government Laws (2018). Bill M 219. 2018 British Columbia Accessibility Act. https://www.bclaws.gov.bc.ca/civix/document/id/lc/billsprevious/3rd41st:m219-1.

[91] Government of Canada Justice Laws Website. Accessible Canada Act (S.C. 2019, c. 10). https://laws-lois.justice.gc.ca/eng/acts/A-0.6/.

[92] British Columbia Technical Bulletins. Information Bulletin Building and Safety Standards Branch. Accessibility in the 2018 British Columbia Building Code of 24 August 2018. https://www2.gov.bc.ca/gov/content/industry/construction-industry/building-codes-standards/bc-codes/technical-bulletins.

[93] Gobierno de España Ministerio de la Presidencia, Relaciones con las Cortes y Memoria Democrática. Agencia Estatal Boletín Oficial del Estado. Real Decreto 732/2019, de 20 de Diciembre, por el que se Modifica el Código Técnico de la Edificación, Aprobado por el Real Decreto 314/2006, de 17 de Marzo. Documento Básico SUA, Seguridad de Utilización y Accesibilidad de 20 de Diciembre de 2019. https://www.boe.es/diario_boe/txt.php?id=BOE-A-2019-18528.

